# Current role of surgery in small cell lung carcinoma

**DOI:** 10.1186/1749-8090-4-30

**Published:** 2009-07-09

**Authors:** Efstratios N Koletsis, Christos Prokakis, Menelaos Karanikolas, Efstratios Apostolakis, Dimitrios Dougenis

**Affiliations:** 1 Department of Cardiothoracic Surgery, School of Medicine, University of Patras, Greece; 2 Department of Anaesthesiology and Critical Care Medicine, School of Medicine, University of Patras, Greece

## Abstract

Small cell lung carcinoma represents 15–20% of lung cancer. Is is characterized by rapid growth and early disseminated disease with poor outcome. For many years surgery was considered a contraindication in Small Cell Lung Cancer (SCLC) since radiotherapy and chemoradiotherapy were found to be more efficient in the management of these patients. Never the less some surgeons continue to be in favor of surgery as part of a combined modality treatment in patients with SCLC. The revaluation of the role of surgery in this group of patients is based on clinical data indicating a much better prognosis in selected patients with limited disease (T1-2, N0, M0), the high rate of local recurrence after chemoradiotherapy with surgery considered eventually more efficient in the local control of the disease and the fact that surgery is the most accurate tool to access the response to chemotherapy, identify carcinoids misdiagnosed as SCLC and treat the Non Small Cell Lung Cancer component of mixed tumors. Performing surgery for local disease SCLC requires a complete preoperative assessment to exclude the presence of nodal involvement. In stage I surgery must always be followed by adjuvant chemotherapy, while in stage II and III surgery must be planned only in the context of clinical trials and after a pathologic response to induction chemoradiotherapy has been confirmed. Prophylactic cranial irradiation should be used to reduce the incidence of brain metastasis

## Background

Small cell lung carcinoma represents 15–20% of all lung cancer and it is basically characterized by rapid growth and early metastatic dissemination. As a result, systemic chemotherapy, with or without radiotherapy, has been typically accepted as the cornerstone of therapy in SCLC [1]. Initially surgery was the treatment of choice for all types of lung cancer but it was abandoned for the subset of SCLC almost 30 years ago after the results of the Medical Research Council (UK) randomized trial which compared radiotherapy and surgery in patients with limited disease [2]. Although the mean survival was less than a year, a small but significant difference in the survival was shown in the two groups of the study: 4 year survival of 3% in the surgery arm and 7% in the radiotherapy arm. A 5 year survival of 5% was noted only in the radiotherapy arm. This report although severely criticized on a number of points, rejected surgery and made radiation therapy the standard form of treatment for many years there after. At about the same period, the first demonstration of the beneficial effects of chemotherapy (CT) (cyclophosphamide vs placebo) was reported by Green et al [3]. They reported a mean survival of 12 weeks with CT compared to only 6 weeks without any treatment. Since that time a great progress has been made in the management of SCLC, taking in account these very poor initial reports. Recently patients with limited disease are treated with curative intent with chemoradiotherapy with a median survival of 23 months and a 5 years survival rate of 12–17%. However, only 30% of all patients with SCLC present limited disease (LD-SCLC). In extensive disease the 5 year survival rate is only 2% [1].

## Materials and methods

The question raised and on which was based this review is whether the adjunction of surgery in the small cell lung carcinoma's management may influence the outcome of specific subset of patients. We proceeded to a review of the Medline PUBMED (January 1980 to Week 1, January 2009) and EMBASE (January 1980 to Week 1, January 2009) literature focusing on retrospective studies where the impact of surgery on survival was evaluated as a stage dependent event. Studies reporting survival rates less than 5 years were excluded. Bibliographies, reference lists of identified studies and review articles were hand-searched. There were no language restrictions. Review Articles and retrospective series from thoracic surgical teams, approaching the surgical management of small cell lung cancer were included. No prospective or randomized controlled trials were found on the issue.

The articles included in this review are representatives of the evolution of the surgical management of small cell lung cancer up to the present.

### Surgery Revived

The role of surgery was revaluated after the introduction of the TNM staging system. The clinical trials that appeared in the literature to argue surgical resection were mostly no randomized and retrospective. Shields et al [4] reviewing the Veterans Administration Surgical Oncology Group experience, postulated that surgery is indicated in LD-SCLC, particularly stage T1, N0 while the issue of limited local recurrence following surgery was pointed out by Shepherd et al from the Toronto group [5]. Similarly the Brompton experience showed 5 year survival rate of 57.1% for stage I [6]. Eventually, these reports revived the interest in the role of surgery in LD-SCLC. Meanwhile adjuvant chemotherapy was evaluated in a more recent report by the Toronto Group and demonstrated an improved survival [7]. There was the need for a prospective randomized trial and indeed this was conducted by the Lung Cancer Study Group. In this trial surgery after induction chemotherapy failed to show a survival improvement or less local recurrence rate compared to radiotherapy [8]. This study has been criticized mainly because patients with T1, N0 disease were excluded from thoracotomy and therefore they were denied of the benefit that radical resection can offer in long term survival. Therefore the role of surgery in the integrated management of small cell lung cancer remained under investigation with retrospective studies demonstrating 5 year survival of approximately 50% for stage I disease [9,10].

### Justification for surgery in early stage SCLC

Anraku and Waddell [11] in a recent excellent review summarized the rational for surgery in SCLC:

1. Small peripheral lung nodules that are in fact typical or atypical carcinoids tumors may be misdiagnosed as SCLC.

2. Histologically mixed tumors with both SCLC and NSCLC components may fail to chemoradiation protocols since there is less sensitivity of the NSCLC component to chemotherapy. Indeed it has been shown that final histology for tumors initially reported as SCLC revealed a NSCLC component in 11–25% [12]. Furthermore studies on neuroendocrine tumors showed that 26.5% of resected SCLC are actually included in the combined small cell lung carcinomas according to WHO more recent classification [11,13]. Thus it seems more logical to offer surgery in mixed or combined small cell tumors.

3. Surgical resection for T1-2, N0, M0 SCLC could offer better local control of the disease compared to chemotherapy alone. Indeed current chemoradiotherapy protocols have demonstrated local failure rates approximating 50% [14]. Additionally R0 surgical resection after induction chemoradiotherapy has shown a control of local relapse in almost 100% of the patients. Likewise, 5 and 10 year survival rates were 39% and 35% for all included patients, resected or not and 44% and 41% respectively for patients with stage IIB to IIIA treated with a trimodality approach including adjuvant surgery [15].

4. Salvage surgery could be preferable compared to second line chemotherapy in cases where after an initial response to chemoradiotherapy a chemotherapy resistant tumor or a local recurrence of the disease has occurred. Similarly patients with mixed histology, as noted above, who present with residual or non responsive tumor after chemoradiotherapy should better be treated with salvage surgery.

5. Second primary, histologically proved NSCLC, tumor after curative chemoradiotherapy for initial SCLC should be surgically resected. Ankaru and Waddell [11] brilliantly emphasized this indication since any new tumor appearing two years after an initial SCLC successfully treated could be a NSCLC.

### Recent evidence supporting the role of surgery

So far there have not been any data from prospective randomized control trials comparing chemotherapy or chemoradiotherapy with induction chemoradiotherapy followed by adjuvant surgery. However accumulated data from non randomized clinical trials have shown that surgery, as part of multimodality treatment protocols, can contribute to both prognosis and local recurrence control (table 1). Granetzny et al [16] in a recent retrospective trial studied the effect of surgery in a trimodality treatment in SCLC. The study included 95 patients, the majority being in stages I and II. Patients were divided in two groups. Group I received surgery followed by adjuvant, mainly platin doublets and anthracycline based, modern chemotherapy protocols. Group II had definitive surgery following neoadjuvant chemotherapy which continued postoperatively in addition to thoracic and cranial radiotherapy. They concluded that patients with stage I and II SCLC can be treated with promising results using a combination of primary surgery and adjuvant chemotherapy as well as thoracic and cranial irradiation. Patients in group II appeared to benefit from lung resection after induction chemotherapy only if complete clearance of mediastinal nodal disease has been achieved, as proven by repeated mediastinoscopy prior to surgical intervention.

**Table 1:** The role of surgery in small cell lung cancer

**Study**	**Protocol-Patients**	**Local relapse**	**Survival**
Fujimori 1997 (22)	P.E based CT × 2–4 → S	5%	Overall MD: 61.9 months (21 pts)
	Patients: 22		3 year: 66.7%
	Resected pts: 21/22		Stage
			I-II: 3 year → 73.3%
			IIIA: 3 year → 42.9%
			p = 0.018

Eberhardt 2003 (13)	Stage IB-IIA: P.E × 4 → S (8 pts)	0%	Overall survival (46 pts)
	Stage IIB-IIIA: P.E × 3 → concurrent CTRx (Hf-RTx) → S (38)		5 year: 39%
			10 year: 35%
	Resected pts: 30/46 (stage IB-IIA:8/IIB-IIIA:22)		Stage IIB-IIIA (22 pts)
			5 year: 44%
			10 year: 41%

Rostad 2004 (15)	CT or concurrent CTRx: 2404 pts		Stage I – 5 year survival
	Surgery: 38 pts		11.3% (CT or CTRx)
	Surgery + additional treatment (CT, RTx, CTRx): 25/38 pts		44.9% (surgery ± additional treatment)

Brock 2005 (19)	S ± adjuvant or induction CT		Stage I
			5 year: 58%
	82 pts		
			Stages II, III and IV
			5 year: 18%, 23%, 0%
			p < 0.001

Tsuchiya 2005 (20)	S → P.E × 4 (62 pts)	10%	Pathological Stage (5 year survival):
	Resected pts: 61/62		I: 73%
			II: 38%
			IIIA: 39%

Granetzny 2006 (14)	S → CTRx (64 pts) – stage I, II		MD
			Primary S: 31,3 months
	CT → S → CT + RTx (thoracic, cranial) (31 pts) – stage IIIA, IIIB		S after CT: 31,7 months (N2-), 12.4 months (N2+)

Bischof 2007 (21)	S → CT ± RTx ± PCI		MD: 47 months
	39 pts: CT 35 pts, RTx 16 pts, PCI 21 pts		1,3, 5 year survival: 97%, 58%, 49%

Lim 2008 (20)	59 pts: 43 pure SCLC, 16 pts:mixed histology		5 year survival: 52%
	Adjuvant therapy: 16/59		T, N, UICC stage not statistically significant

Recent trials supporting the role of surgery in small cell lung cancer (SCLC).

P.E: platinum-etoposside, S: surgery, CTRx: chemoradiotherapy, Hf-RTx: hyperfractionated radiotherapy, CT: chemotherapy, RTx: radiotherapy, MD: median survival.

Rostad et al [17] evaluated 2442 patients with SCLC in a national survey in Norway, the majority of which underwent conventional chemotherapy and concurrent chemoradiotherapy only. Thirty eight patients underwent surgical resection combined with additional modality treatment in 25 of them. For stage I the 5 year survival rate was 11.3% for conventional treated patients compared to 44.9% for patients treated with the addition of surgery. The authors concluded that more patients with peripherally located tumors stage IA and IB should have been referred to surgery.

Similarly in a recent review on the role of surgery in SCLC Leo and Pastorino [18] concluded that surgery can be proposed in T1, T2/N0 disease followed by adjuvant chemotherapy. Surgery in stages II and III must be planned in a multidisciplinary basis, in the context of controlled trials. Likewise, Waddell and Shepherd [19] support the option of surgery in stage I although whether is offered as the initial treatment or after induction therapy remains controversial.

In a recent report by the Imperial College lung resection and mediastinal lymph node dissection used as primary therapy for SCLC either pure (73%) or in mixed histological types was associated with a surprisingly 5 year survival for the total cohort of stage I-III patients of 52% independently of the tumor's T, N and UICC stage [20]. Two issues should be pointed: first, the number of patients receiving adjuvant treatment was small (16 out of 59) and the information on postoperative treatment not robust as stated by the authors and second, there was no separate analysis on survival of patients presenting mixed histological tumors and how the outcome of these patients may have influenced the survival for the total cohort of patients. No matter its limitation this report strongly suggests that selected patients with SCLC, even in more advanced stage disease may benefit from surgery if complete tumor resection is achieved. Moreover this report points on the need to improve the clinical classification models on which the decision making on the management of SCLC patients should be based.

### Patients' selection criteria

It has become clear that precise staging is very important in selecting patients with limited disease. Therefore chest C/T scan, abdominal C/T scan, brain MRI and bone scintigraphy should be included in the evaluation [21]. Modern imaging PET-C/T when available is also mandatory to better define those patients with limited disease. All cases of SCLC who are amenable to surgery, should undergo mediastinoscopy prior to thoracotomy to exclude N2 disease, since these patients who are unlikely to benefit from surgery can be carefully excluded. [8,18] Repeated mediastinoscopy should also be carried out in all cases of induction chemoradiotherapy, where an initial mediastinoscopy has been performed, to determine whether N2 disease still exists. Great effort is given to establish a precise tissue diagnosis prior to surgery. Pathologists should be cautious to rule out any coexisting NSCLC component or a mixed tumor. If SCLC is revealed at frozen section analysis in the operating room then we propose radical resection if multiple frozen sections suggest the absence of hilar or mediastinal nodal involvement. Radical resection and lymph node dissection should also be offered to patients with N2 disease if they can easily tolerate the procedure. Wedge resection should be limited in the less fit patients if hilar or mediastinal lymph node involvement is present.

### Surgery in multimodality treatment with chemotherapy and chemoradiotherapy

Platinum and etoposside based regimens of chemotherapy with concurrent chest radiation followed by prophylactic cranial irradiation (PCI) have reported 5 year survival rates of 22–26% in the twice daily irradiation arm [11,14,22]. In the era of platinum based chemotherapy as adjuvant treatment in patients with LD-SCLC who underwent surgery Brock et al [23] from Baltimore reported a 58% 5 year survival for stage I disease. In a recent study where EP based regimen was used as adjuvant therapy after complete resection without thoracic or cranial irradiation a 73% 5 year survival was noted in the stage IA subset of patients with an overall of 10% of local recurrence rate [24]. The frequency of brain failure however was 15%. This study demonstrated the need of PCI which could prevent brain metastasis [11]. In a recent retrospective analysis Bischof et al [25] reported a median survival of 47 months and 1, 3 and 5 year survival rates of 97%, 58% and 49% respectively in a group of 39 patients undergoing surgery for LD-SCLC. Surgery was followed by platinum based chemotherapy in 90% of the cases, thoracic radiotherapy in 41% of the patients while 21 patients (54%) received PCI. There was a trend towards a better thoracic recurrence free survival and overall survival in the subgroup of patients receiving thoracic irradiation and an improved brain metastasis free survival and overall survival in the subgroup of patients receiving prophylactic PCI. The authors concluded that adjuvant chemotherapy and PCI is necessary in selected patients with LD-SCLC undergoing surgical treatment. Thoracic irradiation should be used in patients with pN1 disease because of an increased risk of subclinical mediastinal lymphatic involvement The best results reported so far have been associated with aggressive trimodality treatment including adjuvant surgery for patients with stage IIB and IIIA the last ones with negative mediastinoscopy prior to surgery [15]. Similar results have been reported by Fujimori et al [26] with a bimodality approach of platinum-etoposside based induction chemotherapy and surgery in stage I-IIIA SCLC with 3 year survival rates of 73.3% for stage I and II and 42.9% for stage IIIA disease. Since the use of chemotherapy and mediastinal irradiation along with surgery seems more than appropriate in the management of selected patients with SCLC the rules on prophylaxis from bronchopleural fistula and the adverse effects of pneumonectomy applied for NSCLC should be also respected in SCLC patients undergoing lung resection. Therefore bronchoplastic lobectomy should be preferred to pneumonectomy when possible and some form of protection of the bronchial stump should be carried out to minimize the risks for stump insufficiency. [27-29]

The ongoing randomized trials in the era of modern chemoradiotherapy regimens may give more definitive answers regarding the precise role of surgery according the stage of the disease [11,30]. Currently there are three ongoing trials of multimodality treatment including surgery for LD-SCLC: the Essen Thoracic Oncology Trial, the West Japan Thoracic Oncology Group and the German Multicenter Randomized Trial (table 2).

**Table 2:** Ongoing trials

**Trial**	**Protocol of treatment**
Essen Thoracic Oncology Group	CT × 3 → concurrent CT + Hf-RTx (45 Gy; twice daily) → Surgery → CTRx

West Japan Thoracic Oncology group	Group I: CT × 3 → concurrent CT + Hf-RTx (45 Gy; twice daily) ± PCI → Surgery
	Group II: concurrent CT + Hf-RTx (45 Gy; twice daily) + CT × 2 ± PCI

German Multicenter Randomised Trial	Group I: CT × 5 → Surgery ± RTx (50 Gy; once daily) + PCI
	Group II: CT × 5 + RTx (50 Gy; once daily) + PCI

Ongoing trials of surgery in a multimodality treatment in SCLC. Adapted by Eberhardt and Korfee (13)

CT: chemotherapy, Hf-RTx: hyperfractionated radiotherapy, RTx: radiotherapy, CTRx: chemoradiotherapy, PCI: prophylactic cranial irradiation

Platinum-etoposside based chemotherapy in all 3 trials.

West Japan Thoracic Oncology Trial: PCI given only in complete remission after induction chemoradiotherapy.

## Conclusion

Despite the lack of scientific evidences based on randomized trials that surgery in limited disease may be superior to chemoradiotherapy [11,18,21,31], we believe that time has come to accept that it has to play an important role either as a primary treatment or as adjuvant therapy, always in the field of multimodality treatment approaches. It is justified to offer primary surgery followed by chemoradiotherapy in stage T1, N0 and possibly in stage T2, N0. In stage II induction concurrent chemotherapy and radiotherapy should be given and radical resection should follow with intent to curative therapy only if there has been a definite initial response to the induction treatment. Prophylactic cranial irradiation should be part of the treatment program only for those patients obtaining a complete remission. In stage IIIA, if adjuvant surgery is planned, a mediastinoscopy should always precede the surgical treatment. If mediastinal clearance has not been achieved then we doubt whether surgery will contribute to survival or local recurrence. Finally surgery should be considered in mixed tumors, as a salvage treatment or in the rare cases of a second NSCLC tumor.

Since a control randomized trial between chemoradiotherapy and primary surgery is difficult to be obtained among patients with limited SCLC, the question how to best integrate surgery into a multimodality approach treatment will remain unclear. We still need to further define and clarify our treatment strategy.

## Competing interests

The authors declare that they have no competing interests.

## Authors' contributions

All authors: 1) have made substantial contributions to conception and design, or acquisition of data, or analysis and interpretation of data; 2) have been involved in drafting the manuscript or revising it critically for important intellectual content; and 3) have given final approval of the version to be published.
